# Characteristics of a responsible community robot: Data from a Delphi study

**DOI:** 10.1016/j.dib.2025.112112

**Published:** 2025-09-27

**Authors:** Ella Maule, Mark Goudswaard, Hemma Philamore

**Affiliations:** University of Bristol, Queen’s Building, University Walk, Bristol, BS8 1TR, UK

**Keywords:** Robotics, Responsible innovation, Citizen science, Consensus, Requirements elicitation, Socio-technical systems, Stakeholder engagement, Participatory design

## Abstract

This article presents data collected during a six-round Delphi study to explore and identify key characteristics that define a robot as a responsible community robot. A total of 20 panellists participated each with relevant technical, organising or contextual expertise.

The study employed a case study of the development of a responsible community robot for a group of citizen scientists to support waterway monitoring, considering the product, process and paradigm of the robot. The panel contributed 170 ideas, which were combined into 111 voting statements and rated by participants from essential to unnecessary.

The dataset presented in this article combines raw questionnaire data from the Delphi study and a record of the intermediate study analysis. The data provided in this article enables further investigation and comparative analyses into the characteristics of responsible community robots across a broader range of contexts. It offers a resource for roboticists and technologists, community groups and other stakeholders interested in the research, development and deployment of responsible community robots.

Specifications Table

**Table:** 

Subject	Engineering & Materials science
Specific subject area	Delphi study to determine design characteristics for responsible, community robotic and autonomous systems.
Type of data	Data Tables, Raw and Processed Supporting materials, including; codebook, text versions of surveys, and text and video supplementary information provided to participants
Data collection	Data were collected through an online survey platform distributed to participating panellists by email invitation. Panellists with relevant expertise from lived experience, practice and/or learned knowledge were recruited by email invitation. For inclusion, panellists were required to be over the age of 18, able to understand and communicate in written and spoken English and have access to a computer or mobile device. No criteria were set for participant exclusion mid-study. The study design was derived from literature and findings from a pilot study.
Data source location	Institution: University of Bristol
Data accessibility	Repository name: Delphi Study Dataset on Responsible Community Robot Characteristics Data identification number: DOI 10.17605/OSF.IO/KBCSR Direct URL to data: https://osf.io/kbcsr/files/osfstorage
Related research article	None

## 1 Value of the Data

• These data are useful in understanding how a multidisciplinary panel of experts conceptualise the defining characteristics of responsible community robotic and autonomous systems, particularly in the context of environmental monitoring.
• The dataset can support researchers and practitioners interested in responsible robotics and technology by providing a set of value-based statements and justifications, developed and evaluated by a multidisciplinary panel through a six-round Delphi study.
• For those involved in the design or evaluation of technologies for communities, these data can inform requirements elicitation processes, identify stakeholder-relevant design considerations, and support benchmarking of value alignment across different contexts.
• For researchers interested in consensus measurement approaches within Delphi studies, these real-world data can be used to examine how different definitions and thresholds of consensus and stability affect the interpretation of expert agreement across multiple rounds.

## 2 Background

This dataset was generated to inform the development of responsible community robotic and autonomous systems (RAS) [1], in response to the need for a clearer articulation of the characteristics that define them.

The research, design, and innovation of robotic and autonomous systems have traditionally occurred within academic and industrial environments, focusing on generating economic value for individual consumers, businesses, or government agencies. By including communities as stakeholders in RAS innovation and taking a broader view of the potential benefits, we can create *Community Robots* that serve community groups in alignment with their values - creating and reinforcing community ties, enabling community action, and prioritising the generation of social value.

Data were gathered using a Delphi study method to facilitate structured, iterative engagement with a diverse, multidisciplinary panel of experts [2]. The study employed a development scenario to provide a concrete context in which to explore the product-, process-, and paradigm-related characteristics of responsible community robots.

## 3 Data Description

**Fig. 1:**
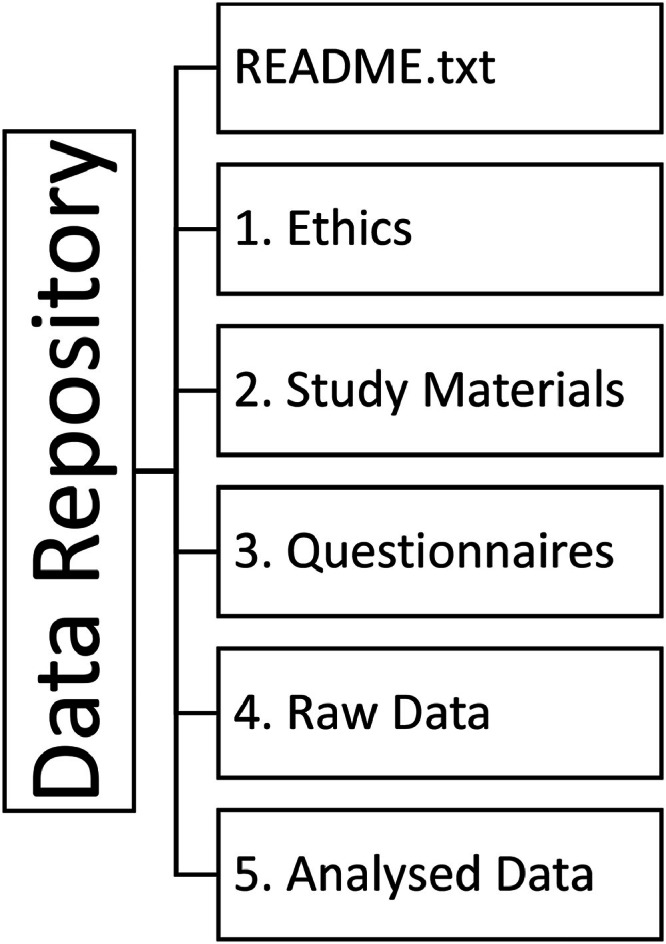
Dataset structure

The *Characteristics of a Responsible Community Robot* dataset [3] contains raw and analysed data, and supporting materials related to the data analyses, study scenario and delivery. The structure of the dataset is shown in Fig. 1. A description of the individual files and their content is provided in Table 1.

**Table 1:** Dataset contents. The REF number is used to map data outputs to relevant methodology steps.

Folder	REF	File Name	Description
		README.txt	Overview of the dataset and folder structure.
1. Ethics	1.1	Participant Information Sheet.odt	Information sheet provided to study participants outlining the purpose and structure of the study.
	1.2	Consent Form.odt	Consent form used to obtain participant agreement to take part in the study.
	1.3	Participation Principles.pdf	
2. Study Materials	2.1	Phase 1 Introduction Video.mp4	Video shown to participants before completing Questionnaire 1.
	2.2	Phase 1 Introduction Transcript.pdf	Transcript of video shown to participants before completing Questionnaire 1.
	2.3	Study Scenario.pdf	Materials provided to participants detailing the study speculative design scenario.
	2.4	Scenario Supporting Information and Definitions.pdf	Materials provided to participants containing additional supporting information.
	2.5	Codebook.odt	The codes used to deductively code the responses during Phase 1 analysis.
	2.6	Phase 2 Introduction Video.mp4	Video shown to participants before completing Questionnaire 3.
	2.7	Phase 2 Introduction Transcript.pdf	Transcript of video shown to participants before completing Questionnaire 3.
	2.8	Phase 2 Voting History Information Video.mp4	Video shown to participants who completed Questionnaire 3 before completing Questionnaire 4.
	2.9	Phase 2 Voting History Information Transcript.pdf	Transcript of video shown to participants who completed Questionnaire 3 before completing Questionnaire 4.
	2.10	Phase 2 Combined Introduction and Voting History Information Video.mp4	Video shown to participants who did not complete Questionnaire 3 before completing their first of Questionnaire 4+.
	2.11	Phase 2 Combined Introduction and Voting History Information Transcript.pdf	Transcript of video shown to participants who did not complete Questionnaire 3 before completing their first of Questionnaire 4+.
3. Questionnaires	3.1	Questionnaire 0.odt	Text version of Questionnaire 0
	3.2	Questionnaire 1.odt	Text version of Questionnaire 1
	3.3	Questionnaire 2.odt	Text version of Questionnaire 2
	3.4	Questionnaire 3.odt	Text version of Questionnaire 3
	3.5	Questionnaire 4.odt	Text version of Questionnaire 4
	3.6	Questionnaire 5.odt	Text version of Questionnaire 5
	3.7	Questionnaire 6.odt	Text version of Questionnaire 6
	3.8	Panel Voting History – Questionnaire 6.ods	Example Voting History document provided to participants in Phase 2
4. Raw Data	4.1	Phase 0_Raw.ods	Raw demographic data from Phase 0
	4.2	Phase 1_Raw.ods	Raw ideation data from Phase 1
	4.3	Phase 2_Raw.ods	Raw voting data from Phase 2
5. Analysed Data	5.1	Phase 0_Analysed.ods	Analysed demographic data from Phase 0
	5.2	Phase 1_Analysed.ods	Analysed ideation data from Phase 1
	5.3	Phase 2_Analysed.ods	Analysed voting data from Phase 2

## 4 Experimental Design, Materials and Methods

**Fig. 2:**
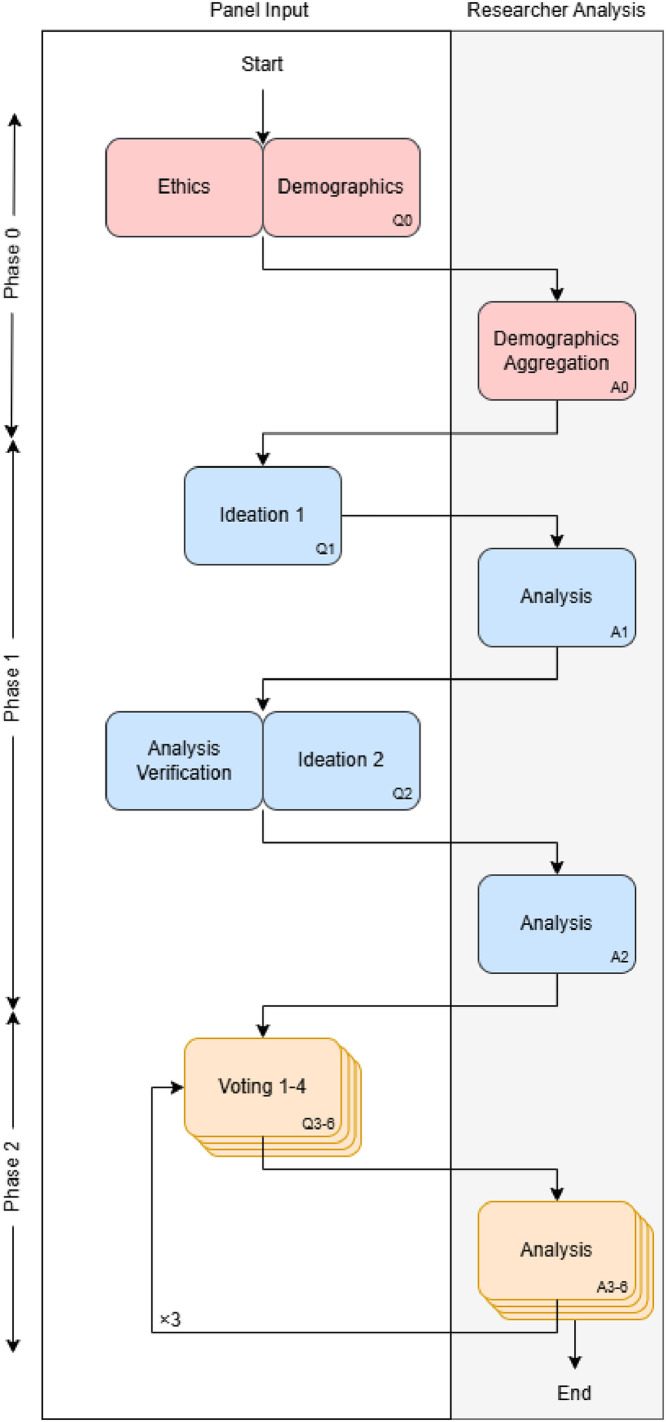
Diagram outlining the Delphi study methodology which took place over three phases. Phase 0: Demographics (one questionnaire), Phase 1: Ideation, (two questionnaires), and Phase 2: Iterative Voting (four questionnaires).

A six-round Delphi study was conducted with 20 participating panelists to explore the characteristics that define a robot as a responsible community robot. The study employed a case study of a waterway monitoring robot to support a community group of citizen scientists. The study was conducted over an eight-month period, using an online survey platform enabling panellists to contribute asynchronously. The methodology described in this section is summarised in Fig. 2.

### 4.1 Study scenario and scope

To investigate the characteristics of a responsible community robot, this study used a study scenario of a waterway monitoring robot for a community group of citizen scientists. The scenario, which was presented to panelists in Questionnaire 1 (Ideation 1), included background on waterway monitoring and citizen science; outlined the robot’s job description; and provided some illustrations of the scenario *(2.3 Study Scenario.pdf)*.

This study asked participating panelists to define the characteristics that make a robot a responsible community robot. They were encouraged to consider the full technology innovation system in their responses, including:

• The Product: The water quality monitoring robot itself.
• The Process: Through which it is developed and used. Including activities like needs assessment, design, prototyping, testing, manufacturing, deployment, repair, maintenance, and end-of-life management.
• The Operating Paradigm: The context in which the robot functions, including its broader impact, the scenarios it enables, and the underlying value system it supports.

### 4.2 Panel selection and recruitment

This study took a broad definition of expert, recognizing and valuing expertise from professional and personal lived experience, practice and learned knowledge equally. A multi-disciplinary panel was recruited with experts identified and recruited according to expertise in one or more of:

• robotics, automation, autonomous systems and digital technology;
• environmental science, monitoring, care, and protection;
• community action, organising; and/or,
• governance and policy.

For inclusion, panellists were required to be over the age of 18, able to understand and communicate in written and spoken English (including the use of any personal assistive devices and accounting for accessibility needs), have access to a computer or mobile device, and have self-evaluated expertise in one or more of the identified disciplinary areas.

Potential panellists were recruited via two methods. First, personalised email invitations were sent to individuals identified through targeted online searches. Second, invitations were disseminated through professional, practice, and interest email group networks. A recruitment target of a minimum of 20 people and a maximum of 35 people was set. For participation, panellists were paid £10 (or equivalent in their local currency) in shopping vouchers per completed questionnaire, paid at the mid-point and end of the study.

### 4.3 Study design

The study was conducted over three phases, consisting of seven questionnaires, including two ideation rounds and four voting rounds. The study design, including stopping criteria, voting statement consensus and stability criteria, and participant exclusion criteria was set *a priori*, informed by literature and the findings of a pilot study [4].

#### 4.3.1 Phase 0: demographics

Upon recruitment, participants were invited to complete Questionnaire 0 *(3.1 Questionnaire 0.odt)* where they were asked to provide demographic information, including age, gender and country of residence. Participants were asked to select the descriptions that best reflect their areas of expertise and to indicate the nature of their experience, the number of years of experience, and any relevant organisations with which they are associated. Additionally, participants were encouraged to provide short reflections on each of the four disciplines of expertise. Aggregated and anonymised panel demographics and reflections were shared with the wider panel in Questionnaire 1 to give the panel an insight and understanding of the diverse expertise and experiences represented.

#### 4.3.2 Phase 1: ideation

The ideation phase comprised two questionnaires. In Questionnaire 1 *(3.2 Questionnaire 1.odt)*, the first ideation round, panelists were first shown a short introductory video presenting the research team, study background, motivation and methodology; and phase activities (*2.1 Phase 1 Introduction Video.mp4, 2.2 Phase 1 Introduction Transcript.pdf)*. Panel members were then provided with key definitions and supporting information relevant to the study *(2.4 Scenario Supporting Information and Definitions)*. The study scenario was introduced, and panelists were invited to respond to the question *“What characteristics define a Waterway Monitoring Robot as a Responsible Community Robot?”,* providing a minimum of four specific characteristics (*2.3 Study Scenario.pdf)*. The responses *(4.2 Phase 1_Raw.ods)* were subsequently analysed to produce a set of refined candidate voting statements *(5.2 Phase 1_Analysed.ods)*. Details on the analysis method are provided in Section 4.5.1.

In Questionnaire 2 (*3.3 Questionnaire 2.odt)*, the second ideation round, the study scenario and question were recapped. Panelists were presented with the combined list of ideas derived from Questionnaire 1 responses *(5.2 Phase 1_Analysed.ods)*, and given the opportunity to contribute any additional characteristics. Panelists who had responded to Questionnaire 1 were asked to review the interpretation of their original contributions and provide clarification where necessary. These clarifications and additional inputs were analysed and incorporated into the finalised list of voting statements. Each statement was assigned a unique three-letter identifier (e.g. [COW]) for reference.

#### 4.3.3 Phase 2: voting

The voting phase comprised four voting rounds, defined *a priori*. In Questionnaire 3 *(3.4 Questionnaire 3.odt)*, the first voting round, panel members were first shown a short introductory video that recapped the study’s purpose, scenario and methodology, and introduced the voting phase *(2.6 Phase 2 Introduction Video.mp4, 2.7 Phase 2 Introduction Transcript.pdf)*. They were then asked to rate the importance of each characteristic (presented in a randomised order) for a waterway monitoring robot to be considered a responsible community robot. This was done on a seven-point Likert scale ranging from Unnecessary to Essential (unnecessary, not at all important, slightly important, moderately important, very important, extremely important, essential). Optionally, participants were invited to provide justification for their votes, to be shared with the wider panel in subsequent rounds. These justifications allowed panelists to clarify or strengthen their position on a voting item. Panelists were encouraged to provide justification to bridge knowledge gaps, highlight relevant examples or scenarios, and identify relevant social and ethical considerations that may influence an item's importance.

The results of this round were analysed *(5.3 Phase 2_Analysed.ods)* and summarized in a voting history document *(*e.g. *3.8 Panel Voting History – Questionnaire 6.ods).* Details on the analysis method are provided in Section 4.5.2 and Section 4.5.3. In Questionnaire 4 (3.5 *Questionnaire 4.odt)*, the second voting round, panelists were shown another short video explaining the analysis methodology and voting history document *(2.8 Phase 2 Voting History Information Video.mp4, 2.9 Phase 2 Voting History Information Transcript.pdf)*. In Questionnaires 4 to 6 *(3.5 Questionnaire 4.odt, 3.6 Questionnaire 5.odt, 3.7 Questionnaire 6.odt)*, voting rounds two to four, panelists voted on the statements that had not yet reached the consensus or stability threshold in previous voting rounds. The statements were presented in ascending order of agreement. In these rounds, panelists were provided with a voting history document containing descriptive statistics and justifications for each statement, summarizing the panel position. The descriptive statistics provided included a measure of dispersion, a central tendency indicator and a frequency count for each Likert rating. Voting statements were grouped by status as either Open, At Consensus, or Stable. Where data were available, documents were personalised to include each panelist’s previous round votes.

### 4.4 Data collection

All data were collected using the online survey platform Qualtrics. Upon publication of each questionnaire, participants were invited by email to complete the survey at their convenience within a minimum two-week response window. The questionnaires could be completed in parts, revisited as needed within this time. Midway through the completion window, a reminder email was sent to panellists who had not yet responded. To ensure data quality, attention checks were included in voting rounds with >30 statements, with failed checks resulting in data exclusion from further analysis.

### 4.5 Data analysis

#### 4.5.1 Phase 1: qualitative analysis of ideated voting statements

Responses contributed during Phase 1 were first segmented into independent clauses, each representing a distinct and self-contained idea. These were then deductively coded, assigning relevant codes from the codebook.

The codebook was developed using an abductive approach based upon findings from a pilot study previously conducted by the authors [4,5]. Codes that were inductively generated in the pilot study were first refined. These refined codes were subsequently compared and mapped against established engineering design and quality frameworks, including Garvin’s dimensions of quality [6] and Pugh’s Design Specification [7]. Through an iterative process of revising the codebook and reviewing the pilot study data, a final set of 28 codes and definitions was produced *(2.5 Codebook.odt)*.

Once coded, items were reviewed by thematic code. Responses were grouped and consolidated to eliminate repetition and standardize format, while preserving important distinctions. Where necessary, statements were rephrased to be specific, descriptive and actionable. The impact and contribution of each participant statement was tracked using statement unique identifiers *(5.2 Phase 1_Analysed.ods)*.

#### 4.5.2 Phase 2: quantitative analysis of voting results

The Panel’s votes were analysed to evaluate the degree of agreement as defined by (1) a measure of dispersion and (2) the central tendency.

The measure of dispersion was evaluated by Tastle and Weirman’s Consensus Measure (*C_m_*) [8]:

$$C_{m}\left(X\right)=\sum_{i=1}^{n}p_{i}\log_{2}\left(1-\frac{\left|X_{i}-\mu_{X}\right|}{d_{X}}\right)$$

A consensus threshold of $C_{m}\geq 0.8$, and stability threshold of $\Delta C_{m}\leq 0.04$ were used, informed by the results of the pilot study [4,5]

The central tendency was assessed using Tastle and Weiman’s Agreement measure (*A_m_*) [9], reported as the Likert rating (τ) corresponding to the maximum value of *A_m_*.

$$A_{m}\left(X,\tau \right)=\sum_{i=1}^{n}p_{i}\log_{2}\left(1-\frac{\left|X_{i}-\tau \right|}{2d_{X}}\right)$$

The thresholds for consensus and stability were determined via a pilot study [4,5]. Consensus was defined as $C_{m}\geq 0.8$, while stability was defined as $\Delta C_{m}\leq 0.04$ between subsequent rounds, provided the Likert central tendency $\tau_{A}$ remained the same.

**Fig. 3:**
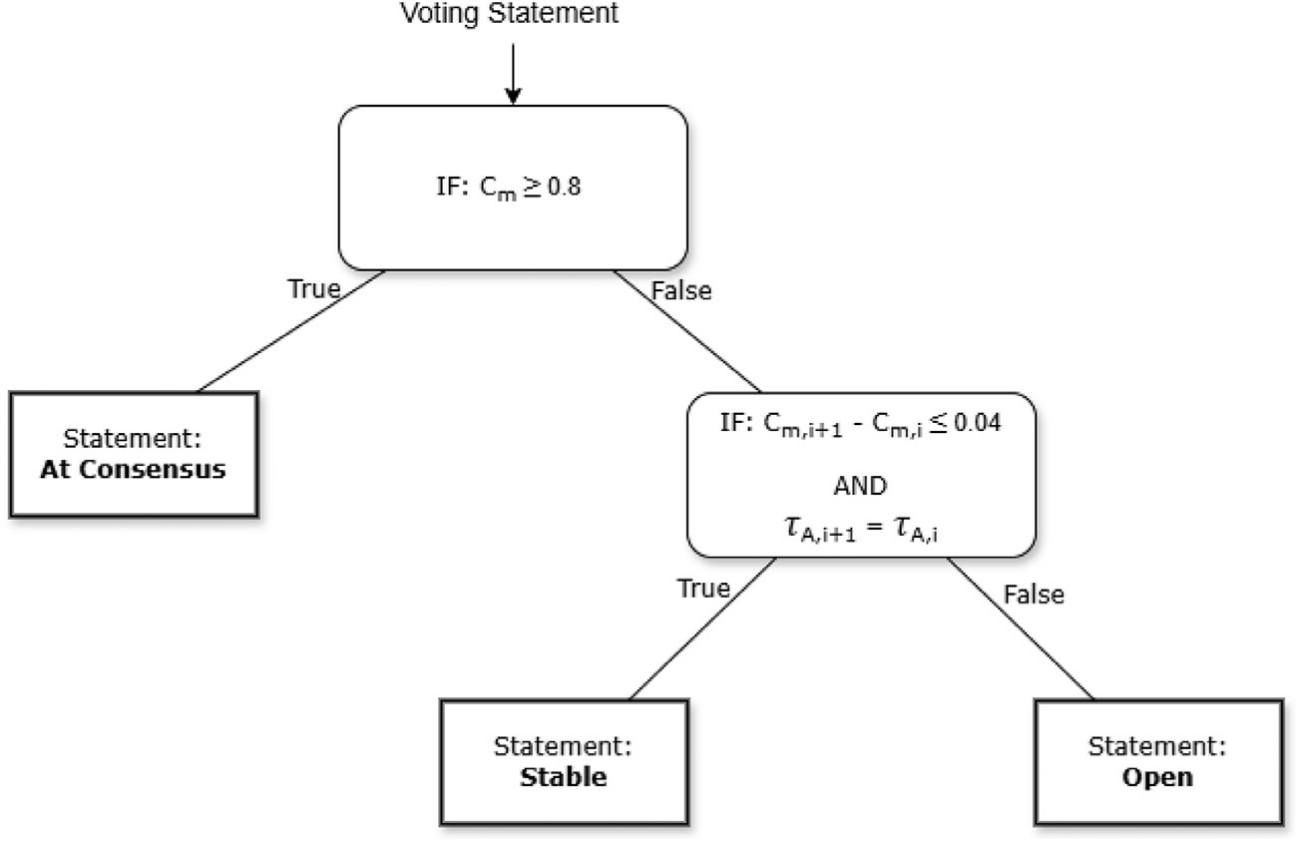
The decision tree used to evaluate the status of voting statements after each voting round.

For each voting statement, the result of the analysis was used to determine its status, as either *Open, At Consensus*, or *Stable,* going into the next voting round. Items evaluated as *Open* were carried forward for further voting, while items evaluated as *At Consensus* or *Stable* were frozen. The process and criteria to evaluate each item’s status are presented in a flow chart in Fig. 3 below.

Out of the 111 Voting Statements, 75 achieved consensus, 33 were stable, and 3 remained open by the close of voting round 4 (Q6).

#### 4.5.3 Phase 2: qualitative analysis of vote justifications

Justifications were reviewed for their relevance and anonymity. For each voting statement, all relevant statements from previous rounds were aggregated and included in the Panel Voting History document.

## Limitations

Several limitations associated with the study data should be considered. Firstly, a limited panel size of 20 participants took part in the study, with not all participants taking part in each round. Consequently, only the views and contributions of those participating in each round were included in the intermediate analysis.

Secondly, the data were collected over an eight-month period, introducing the possibility of external factors influencing participants' opinions. The extent to which these external influences, as opposed to the provided voting history information, shaped the outcomes remains unknown.

Finally, the development scenario used in this study presents a further limitation to the generalisability of the data. The nature of the scenario will inevitably have influenced the voting statements generated by the panellists, and as such, they should be considered incomplete. The impact of the scenario on the results of the voting rounds is, however, unknown.

## Ethics Statement

Informed consent was obtained from all participants of the study. The study was granted ethical approval by the University of Bristol Faculty of Engineering Research Ethics Committee, ref: 20,527.

## CRediT Author Statement

**Ella Maule:** Conceptualization, Methodology, Investigation, Formal Analysis, Resources, Data Curation, Writing - Original Draft, Writing - Review & Editing, Visualization, Project administration; **Mark Goudswaard:** Methodology, Writing - Review & Editing, Supervision; **Hemma Philamore:** Conceptualization, Methodology, Writing - Review & Editing, Supervision.

## Data Availability

Open Science FrameworkDelphi Study Dataset on Responsible Community Robot Characteristics (Original data).
